# Isoindigo-Based Small Molecules with Varied Donor Components for Solution-Processable Organic Field Effect Transistor Devices

**DOI:** 10.3390/molecules200917362

**Published:** 2015-09-18

**Authors:** Hemlata Patil, Jingjing Chang, Akhil Gupta, Ante Bilic, Jishan Wu, Prashant Sonar, Sheshanath V. Bhosale

**Affiliations:** 1 School of Applied Sciences, RMIT University, GPO Box 2476, Melbourne Victoria 3001, Australia; E-Mail: hemlatap2@gmail.com (H.P.); 2 Institute of Materials Research and Engineering (IMRE), A*STAR (Agency for Science, Technology and Research), 3, Research Link, Singapore 117602, Singapore; E-Mails: chjj1234@gmail.com (J.C.); chmwuj@nus.edu.sg (J.W.); 3 Medicinal Chemistry, Monash Institute of Pharmaceutical Sciences, Monash University, Parkville Victoria 3052, Australia; 4 CSIRO Manufacturing, Virtual Nanoscience Lab, Parkville Victoria 3052, Australia; E-Mail: ante.bilic@csiro.au; 5 School of Chemistry, Physics and Mechanical Engineering, Queensland University of Technology, GPO Box 2434, Brisbane QLD 4001, Australia

**Keywords:** isoindigo, solution-processable, organic field effect transistors, donor-acceptor-donor, carbazole, triphenylamine

## Abstract

Two solution-processable small organic molecules, (*E*)-6,6′-bis(4-(diphenylamino)phenyl)-1,1′-bis(2-ethylhexyl)-(3,3′-biindolinylidene)-2,2′-dione (coded as **S10**) and (*E*)-6,6′-di(9*H*-carbazol-9-yl)-1,1′-bis(2-ethylhexyl)-(3,3′-biindolinylidene)-2,2′-dione (coded as **S11**) were successfully designed, synthesized and fully characterized. **S10** and **S11** are based on a donor-acceptor-donor structural motif and contain a common electron accepting moiety, isoindigo, along with different electron donating functionalities, triphenylamine and carbazole, respectively. Ultraviolet-visible absorption spectra revealed that the use of triphenylamine donor functionality resulted in an enhanced intramolecular charge transfer transition and reduction of optical band gap, when compared with its carbazole analogue. Both of these materials were designed to be donor semiconducting components, exerted excellent solubility in common organic solvents, showed excellent thermal stability, and their promising optoelectronic properties encouraged us to scrutinize charge-carrier mobilities using solution-processable organic field effect transistors. Hole mobilities of the order of 2.2 × 10^−4^ cm^2^/Vs and 7.8 × 10^−3^ cm^2^/Vs were measured using **S10** and **S11** as active materials, respectively.

## 1. Introduction

Over the past two decades, the design and development of new organic semiconductors has been a subject of increasing research interest since these materials are widely used as active components for electronic devices such as light emitting diodes [1], field effect transistors [2], photodiodes [3], and photovoltaic cells [4,5,6,7,8]. Such vast and industrial applications place high demands on both the electronic and chemical properties of materials, including chemical and thermal stability, broad absorption profile, appropriately tuned energy levels, solution-processability and charge carriers’ mobility. The fulfilment of such properties constitutes a challenge for synthetic organic chemists who generally use a “structural strategy” in which blocks of distinctive electronic properties are assembled together in one chromophore through appropriate chemical coupling reactions [9].

Prior to their use as active materials for organic electronic devices, semiconductor materials were also used for optical data storage or optical switching [10,11]. Such materials/structures are typically based on a “push-pull” modular design that combines electron-rich (donor) and electron-deficient (acceptor) units connected via a π-conjugated linker [8,12]. Such designs have turned out to be highly successful in view of generating new semiconducting materials, controlling highest occupied molecular orbital (HOMO) and lowest unoccupied molecular orbital (LUMO) energy levels of target chromophores and tuning optoelectronic and redox properties. This approach has also been successful in tuning the solubility of target materials which is an essential requirement for the fabrication of organic electronic devices. These push–pull or donor–acceptor (D–A) modules allow an intramolecular charge transfer (ICT) transition that is beneficial for broadening absorption spectrum and narrowing the optical band gap. Most studied designs with various D–A combinations include, but are not limited to, D–A, D–A–D, A–D–A and A–D–A–D–A. Because of the vast majority of structural blocks and advances in organic chemistry, it is not surprising that there is tremendous interest in the development of such designs by varying D–A combinations with the goals of achieving panchromatic absorbance, appropriate energy levels and solution processability. Exploration of such designs is possible with polymeric entities as well as small organic molecules. However, small molecular semiconductors can offer advantages over polymeric counterparts in terms of ease of synthesis, introduction of structural variation, purification, less batch to batch variations and less end-group contamination. It has been realized that in order to take full advantage of the properties of small organic molecules, engineering of chemical structure as well as incorporation of appropriate D and/or A functionalities are highly desirable [13,14]. We are decidedly interested in exploring small organic molecules for their potential applications in organic electronics.

In our efforts to design and develop versatile and novel materials for organic electronic applications [15,16,17,18], we are interested in exploring the D–A–D module in particular. We [19] and others [20,21,22,23] have demonstrated that such modules facilitate the favorable π-π interactions in the film, leading to an enhanced charge transport between adjacent molecules. Few examples of acceptor functionalities within a D–A–D structural motif include diketopyrrolopyrrole (DPP), 2-pyran-4-ylidenemalononitrile, thiazolothiazole, naphthalene diimide (NDI) and isoindigo [20,21,22,23,24,25,26,27]. The use of isoindigo has been widely reported to develop polymeric entities for organic electronics [28,29], however, progress on small molecular semiconductors is finitely reported [8]. A material development program based on isoindigo functionality has trailed behind other emerging accepting functionalities.

**Figure 1:**
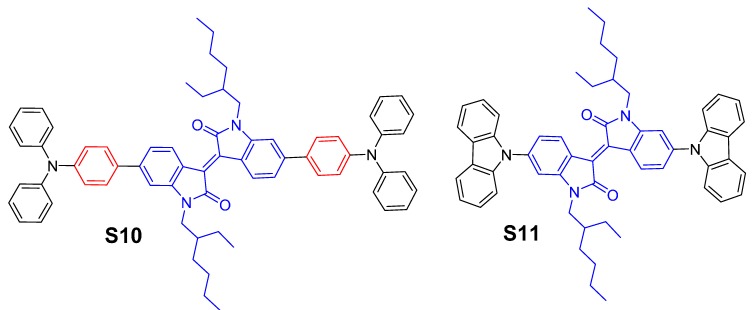
Molecular structures of the new organic materials investigated in this work.

In this study, we report the design, synthesis and characterisation of the optoelectronic properties of two new materials—(*E*)-6,6′-bis(4-(diphenylamino)phenyl)-1,1′-bis(2-ethylhexyl)-(3,3′-biindolinylidene)-2,2′-dione (**S10**) and (*E*)-6,6′-di(9*H*-carbazol-9-yl)-1,1′-bis(2-ethylhexyl)-(3,3′-biindolinylidene)-2,2′-dione (**S11**)—which are based on isoindigo functionality and are represented in Figure 1. The chemical structures of both the materials, **S10** and **S11**, were confirmed by ^1^H- and ^13^C-NMR spectroscopies and high resolution mass spectrometry. The target materials prepared in this work were found to be highly soluble in a variety of common organic solvents, such as dichloromethane, chloroform, chlorobenzene and toluene, which is a feature that is essential for the fabrication of solution-processed organic semiconductor devices. For instance, the solubility of **S11** was found to be as high as 15 mg/mL of chloroform whereas **S10** exerted better solubility at 22 mg/mL of chloroform, thus indicating that triphenylamine functionality can enhance the solubility profile of a target chromophore when compared with an analogue. We have fabricated the solution-processable organic field effect transistors (OFETs) using **S10** and **S11**. Hole mobilities of the order of 2.2 × 10^−4^ and 7.8 × 10^−3^ cm^2^/Vs were measured using **S10** and **S11**, respectively. This study builds upon our search for the versatile and efficient organic materials by exploring D–A–D module and is a comparative study of the effect of different donors (triphenylamine (**S10**) and carbazole (**S11**)) whilst keeping the acceptor part (isoindigo) constant.

## 2. Results and Discussion

### 2.1. Design Strategy, Synthesis and Characterisation

**Scheme 1:**
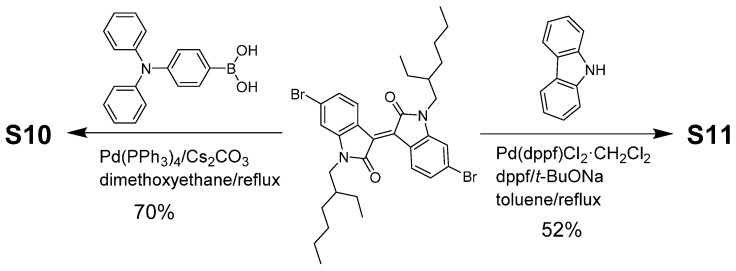
Reaction scheme for the synthesis of **S10** and **S11**.

Both materials, **S10** and **S11**, were developed based on the D–A–D module, and the central acceptor moiety was directly linked to the donor functionalities to create a highly conjugated backbone. The development of such structures involves the use of two identical donor units placed on either side of the central core, thus resulting in a symmetrical chromophore. In **S10**, triphenylamine (TPA) group was selected for its believed ability to act as an energy antenna [30], which may be responsible for an overall bathochromic absorption when compared with **S11** for which carbazole functionality was used. Both **S10** and **S11** were synthesized as per reaction Scheme 1 (For their spectra, please see Supplementary Figures S1–S8 in Supplementary Materials). The use of isoindigo functionality for enhancing the solubility of target materials is paramount as it allows an incorporation of lypophilic chains on its core nitrogen atoms. These alkyl chains facilitate the deposition of target materials as films or layers on appropriate substrates by relatively simple fabricating techniques.

### 2.2. Optoelectronic Properties

**Figure 2:**
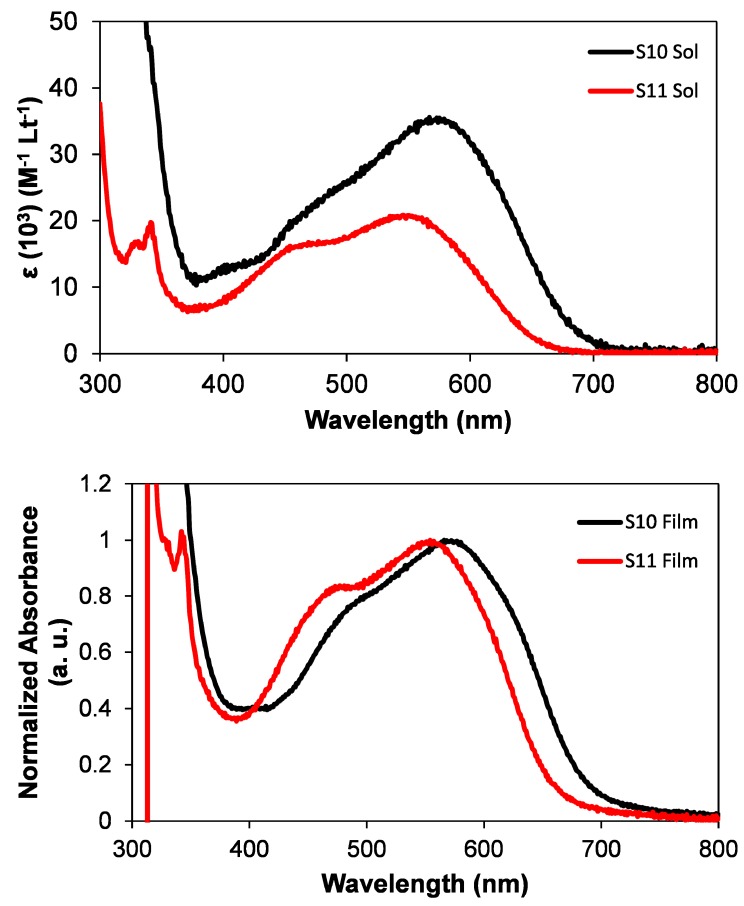
Molar absorptivity of newly synthesized materials **S10** and **S11** in chloroform solutions (upper) and normalized UV–Vis absorption spectra of **S10** and **S11** in thin solid films (lower), spin-cast from their chloroform solutions (equimolar solutions of **S10** and **S11** spun at 2500 rpm for 1 min).

The optical properties of **S10** and **S11** were investigated by measuring their ultraviolet-visible (UV-Vis) absorption spectra in chloroform solution and in pristine as-casted films (Figure 2). The longest wavelength absorption maximum (λ_max_) exhibited by **S10** was at 572 nm with an absorption onset at 710 nm. **S11** exhibited its λ_max_ at 560 nm with an onset at 670 nm. Both the absorption maximum and extinction coefficient increased with the use of TPA donor unit. This is in agreement with the design principle that the use of TPA donor unit can indeed act as an energy antenna that is helpful for stronger bathochromic shift when compared with other donor functionalities, such as carbazole. This is consistent with the idea that greater ICT can be achieved with increasing donating strength. Absorption spectra of **S10** and **S11** in pristine films were also measured by spin-casting their films from chloroform solutions (equimolar solutions of **S10** and **S11** spun at 2500 rpm for 1 min). Thin film absorption spectra exhibited bathochromic shifts when compared with solution spectra, a finding that is consistent with literature reported materials [15,16]. The use of TPA donor unit in **S10** provided an augmentation of λ_max_ by 20 nm when compared to the carbazole unit in **S11**. Evidently, the use of such a strong donor can be helpful (1) to enhance the absorption profile of a given chromophore that can lead to greater light harvesting on its surface during the process of photo-excitation and (2) to intensify the π-conjugation within the molecular backbone.

**Figure 3:**
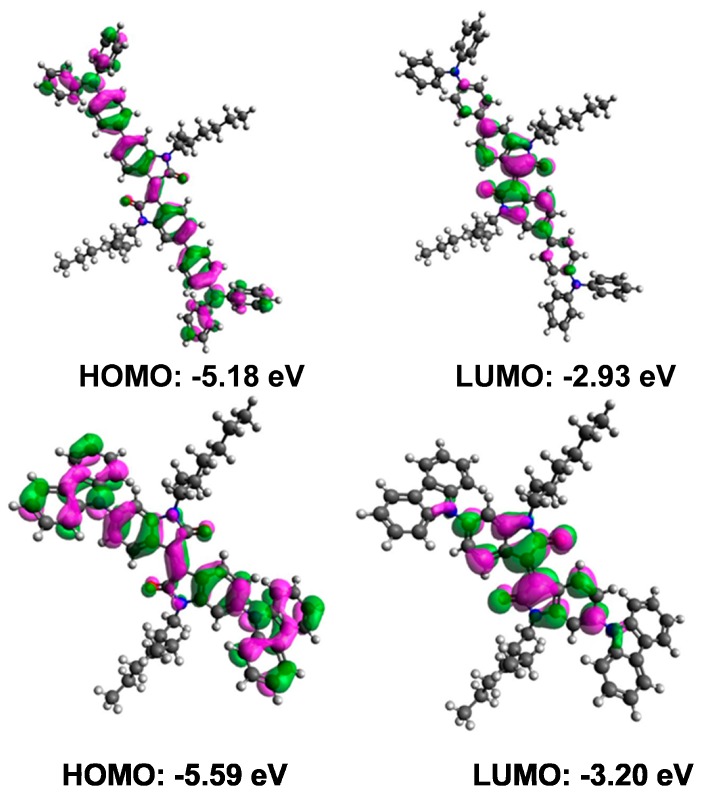
Orbital density distribution for the HOMOs and LUMOs of **S10** (upper) and **S11** (lower). DFT calculations on both the materials were performed using the Gaussian 09 software suite and the B3LYP/6-311+G(d,p)//B3LYP/6-31G(d) level of theory.

We performed density functional theory (DFT) calculations on both the materials using the Gaussian 09 suite of programs [31] and B3LYP/6-311+G(d,p)//B3LYP/6-31G(d) level of theory. DFT investigation indicated that HOMO to LUMO excitation moves the orbital density distribution from the donor functionalities to the isoindigo core unit. The HOMO densities of **S10** and **S11** have spread across the whole molecular backbone with major dwelling over the donor functionalities. The LUMO orbital densities were delocalized over the central part of the molecule and received a sizable contribution from the isoindigo core unit (see Figure 3). This type of density distribution may be advantageous for ICT transition between donor and acceptor components. It was further observed that the use of a TPA unit as an energy antenna in **S10** improves the electron-donating ability and the theoretical optical band-gap was reduced as a result of using TPA (2.26 eV (**S10**)) against carbazole functionality (2.39 eV (**S11**)). Experimentally, the HOMO energies of **S10** and **S11** were estimated using photoelectron spectroscopy in air (PESA) and the LUMO energies were calculated by adding the band gap determined by the onset of thin film UV–Vis absorption to the HOMO values.

**Figure 4:**
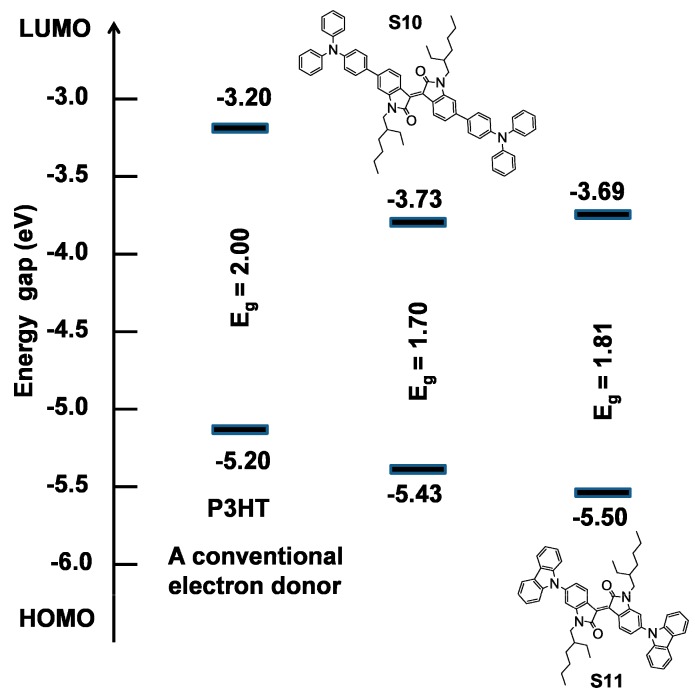
Energy level diagram depicting HOMOs LUMOs of **S10** and **S11**, where HOMO levels were measured using PESA on thin solid films and LUMO levels were calculated from the optical band gaps and HOMO levels (E_LUMO_ = E_bandgap_ + E_HOMO_).

These PESA measurements were performed on thin solid as-casted films (same films that were used to measure the absorption spectra) to measure work functions corresponding to their HOMO levels. The HOMO energy level of **S10** was reduced by 0.07 eV in comparison to the HOMO level of **S11**. The band gap was reduced by 0.11 eV with the use of a TPA donor in **S10**. These experimental findings (1) followed the theoretical calculations trend which indicated that with the use of a strong donor, of which TPA is an example, band gap reduces and (2) that both the target chromophores are electron donating semiconducting components (see energy level diagram, Figure 4).

**Figure 5:**
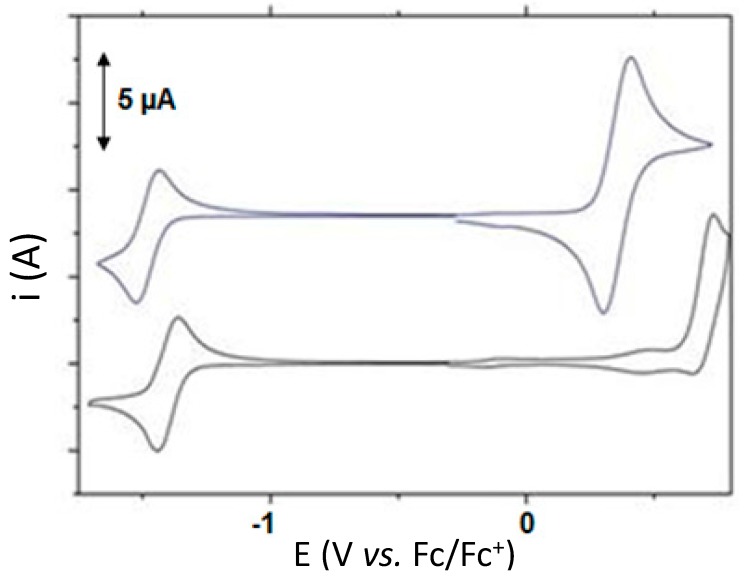
Cyclic-voltammograms of **S10** (upper) and **S11** (lower), run in freshly distilled dichloromethane at a sweep rate of 50 mV·s^−1^, showing reversible reduction potential waves (both **S10** and **S11**) and reversible oxidation potential wave (**S10**).

Apart from PESA measurement, we also conducted the cyclic-voltammetry (CV) experiments in order to observe the solution behavior of these materials, and to look out for reversible oxidation potentials (one or multiple) which might suggest the suitability of these materials to be used as donor semiconducting components. We conducted the CV experiments on a glassy carbon electrode. The cyclic voltammograms are included in Figure 5. Both the compounds exhibit a chemically reversible first cathodic process, thus indicating that the species formed by acceptance of an electron is stable on the voltammetry timescale. Direct connection of TPA donor to the diimide core of **S10** pushes the first reduction process to a more cathodic potential relative to **S11**, resulting in a higher LUMO of −3.47 eV for **S10** (calculated by the onset potential method relative to ferrocene) compared to **S11**, which has the LUMO energy of −3.55 eV. This suggests that the direct bond of tertiary nitrogen atom to the central π-system in **S11** reduces the electron density of electro-active centre of **S11** compared to **S10**. Reversible oxidation potential in **S10** indicates the strong donating capacity of TPA donor, an observation that verifies the design principle, absorption behavior as well as literature reports [15].

**Figure 6:**
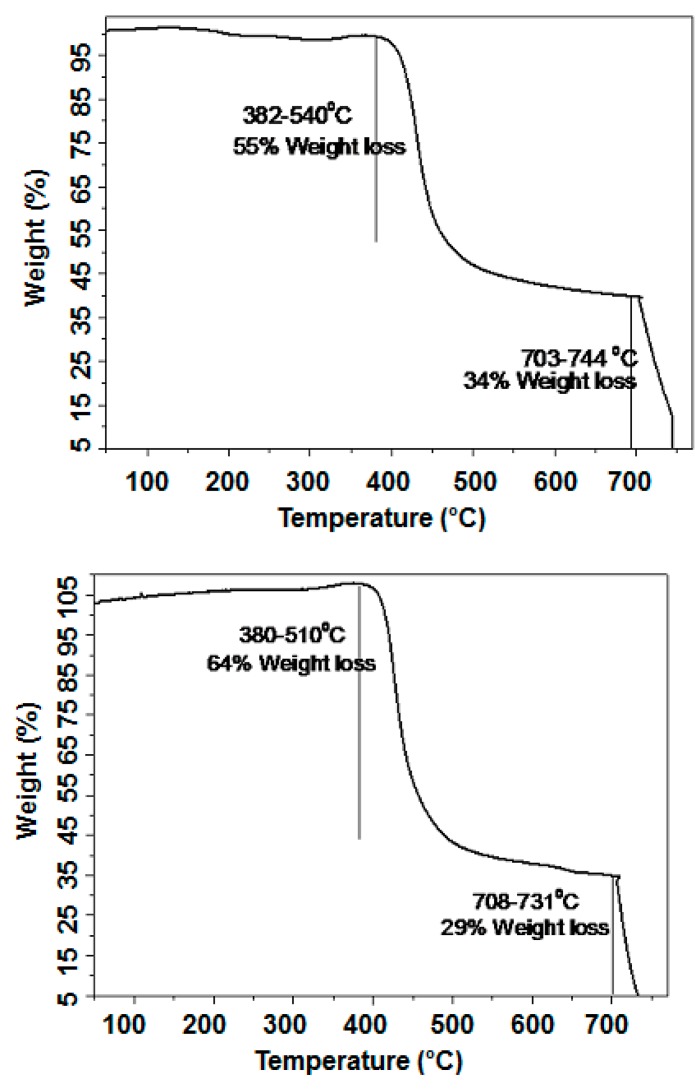
TGA traces of **S10** (upper) and **S11** (lower) under nitrogen atmosphere. Heating rate: 10 °C/min from room temperature to 800 °C.

It was further realized that despite the presence of intriguing and advantageous optoelectronic properties, organic semiconducting materials must possess thermal stability so that they can sustain rigid device fabricating conditions, such as device annealing at a higher temperature. In line with this requirement, we conducted thermogravimetric and differential scanning calorimetry analyses. Thermogravimetric analysis (TGA) curves of **S10** and **S11** were run at a heating rate of 10 °C min^−1^ under the protection of nitrogen. TGA indicated that both **S10** and **S11** are thermally stable up to 350 °C (Figure 6), a finding that supports high temperature annealing of as-casted organic electronic devices and corroborates differential scanning calorimetry (DSC) analysis (Supplementary Figure S9).

**Figure 7:**
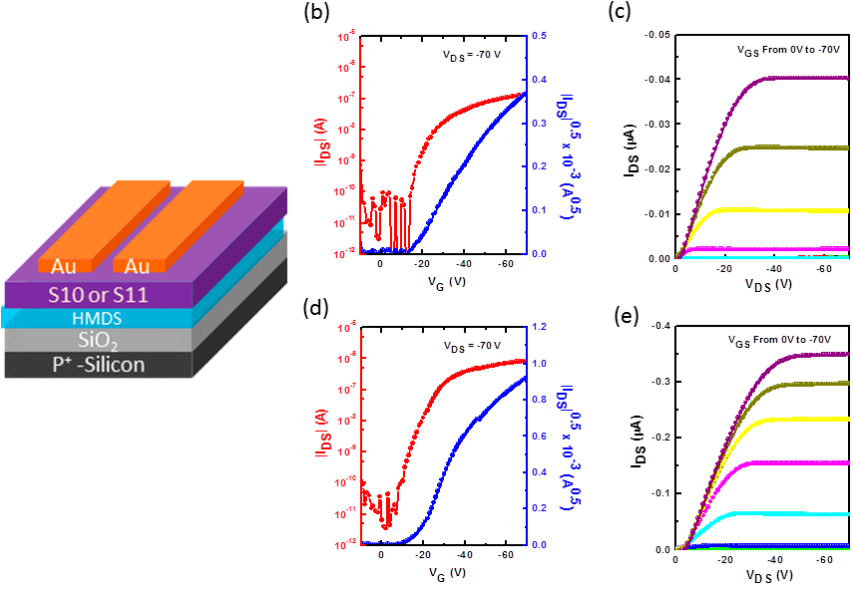
(**a**) Organic field effect transistor (OFETs) device geometry. Output and transfer characteristics of **S10** (**b**,**c**) and **S11** (**d**,**e**) based p-channel OFET annealed at 120 °C on HMDS treated *n*^+^-Si/SiO_2_ substrate. The hole transfer curves were derived at drain voltages (V_D_) of −70 V.

The electrical properties of **S10** and **S11** as an active channel semiconductor in OFET devices were characterized using a bottom-gate, top-contact geometry. Heavily *n*-doped conductive silicon wafer with a layer of ~200 nm SiO_2_ on the surface was used as the substrate. The SiO_2_ functions as the gate insulator and the doped Si as the gate. The active **S10** and **S11** thin films (~40 nm) were spin coated on top of the HMDS modified SiO_2_ surface using **S10** and **S11** CHCl_3_ solution (0.5 wt %). On top of **S10** and **S11** thin films, gold was deposited as source and drain electrode via shadow mask. The OFET schematic of the complete device fabrication is shown in Figure 7a. The small molecule thin films were selectively annealed at 100 °C and 120 °C for 10 min on a hot plate in nitrogen atmosphere. OFET devices exhibit typical p-type electrical characteristics. The hole mobility was calculated from the saturation regime of transfer curve. The **S10** and **S11** thin film annealed at 120 °C exhibited hole mobility of 2.2 × 10^−4^ and 7.8 × 10^−3^ cm^2^/Vs, respectively (Table 1). The transfer and output characteristics of 120 °C annealed **S10** and **S11** based OFET devices are shown in Figure 7b–e, respectively. The on/off ratios for all of the devices were calculated around 10^4^ to 10^5^ whereas the threshold voltage was observed in the range of −21 to −16 V with respect to annealing. It should be noted that the interface between semiconductor and electrodes as well as the dielectric and semiconductor are critical for charge carrier injection and transport properties. Among them, the dielectric interface is much more crucial, and the charge carrier traps caused by silanol groups on dielectric surface could affect the electron transport more significantly. The radical anions formed are unstable towards water and oxygen due to their low LUMO energy levels (around −3.7 eV), and can be easily trapped at the interface. Therefore, in our studies, only hole transport behavior was observed for these two compounds. To our knowledge, there are very few reports which explore isoindigo-based small molecules and their applications in OFET devices. The obtained hole mobility values for **S10** and **S11** are in good agreement with some of the previously reported small molecules based on isoindigo core [32].

**Table 1:** OFET device performance of **S10** and **S11** thin films annealed at 100 °C, and 120 °C on HMDS treated *n*^+^-Si/SiO_2_ substrates using bottom-gate, top-contact (BGTC) device architecture.

Annealing Temperature (°C)		μ (cm^2^·V^−1^·s^−1^)	V_T_ (V)	On/Off Ratio
**S10**	100	1.0 × 10^−5^	−18–−25	2.5 × 10^3^
	120	2.2 × 10^−4^	−16–−20	1.1 × 10^4^
**S11**	100	8.5 × 10^−4^	−20–−24	4.8 × 10^4^
	120	7.8 × 10^−3^	−17–−21	1.3 × 10^5^

**Figure 8:**
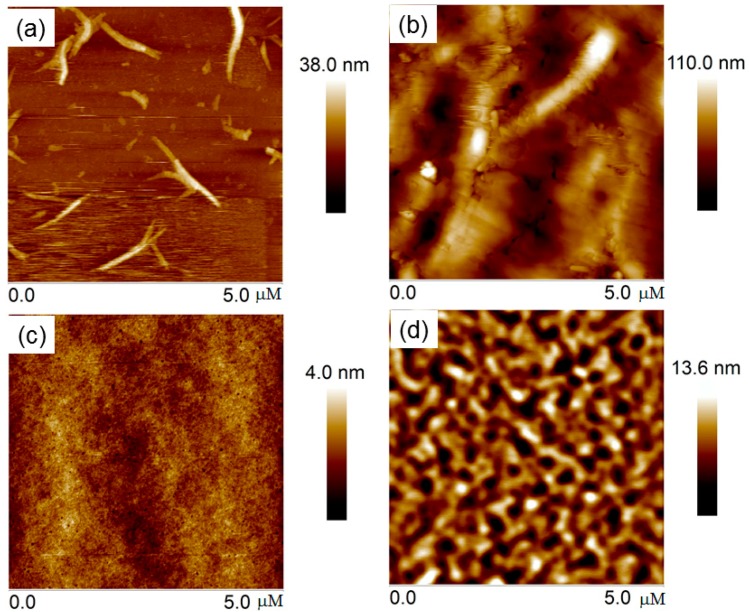
The AFM images of **S10** (**a**,**b**) and **S11** (**c**,**d**) for as-cast and thermally annealed films.

The surface morphologies of **S10** and **S11** thin films were studied by atomic force microscopy (AFM) in the tapping mode and are shown in Figure 8. The spin coated films exhibited amorphous domains for both the materials. For **S10**, upon thermal annealing at 120 °C, the thin films become discontinuous due to strong aggregation, which deteriorates the charge transport properties. Compared to **S10**, **S11** exhibited an interconnected network, which is beneficial for charge carrier transport. Two dimensional X-ray diffraction measurements of these two films were further investigated in order to study the crystallinity and microstructure of these films (see Supplementary Figure S10). It can be seen that both thin films exhibited week diffraction peaks. After thermal annealing, the diffraction intensity was enhanced for **S10**, which is consistent with the AFM image. However, for **S11**, the thin film still showed weak diffraction peak after thermal annealing, indicating its amorphous nature. These results are consistent with the results of surface morphologies as well as slight low charge transport properties.

## 3. Experimental Section

### 3.1. Materials and Instruments

All the reagents and chemicals used, unless otherwise specified, were purchased from Sigma-Aldrich Co. (Sydney, Australia). The solvents used for reactions were obtained from Merck Specialty Chemicals (Sydney, Australia) and were used as such. (*E*)-6,6′-dibromo-1,1′-bis(2-ethylhexyl)-(3,3′-biindolinylidene)-2,2′-dione was purchased from Luminescence Technology Corporation (LTC, Taiwan) and was used as such. Unless otherwise specified, all ^1^H- and ^13^C-NMR spectra were recorded using a Bruker AV400 spectrometer (Bruker Corporation, Billerica MA, USA) at 400 MHz and 100.6 MHz, respectively. Chemical shifts (δ) are measured in parts per million (ppm). Thin layer chromatography (TLC) was performed using 0.25 mm thick plates precoated with Merck Kieselgel 60 F_254_ silica gel (Merck, Darmstadt, Germany), and visualized using ultraviolet (UV) light (254 nm and 365 nm, Spectronics Corporation, Westbury, NY, USA). Melting points were measured using a Gallenkamp MPD350 digital melting point apparatus (Sanyo, Osaka, Japan) and are uncorrected. High-resolution mass spectra (atmospheric-pressure chemical ionization (APCI)) experiments were performed with a thermo scientific Q *E*xactive Fourier-transform mass spectrometer (Thermo Scientific, Bremen, Germany), ionizing by APCI from an atmospheric solids analysis probe (ASAP) [33]. UV-Vis absorption spectra were recorded using a Hewlett Packard HP 8453 diode-array UV-Vis spectrometer (Agilent Technologies, Mulgrave Victoria, Australia). Work functions of all the materials were estimated using PESA. PESA measurements were recorded using a Riken Keiki AC-2 PESA spectrometer (RKI Instruments, Union City, CA, USA) with a power setting of 5 nW and a power number of 0.5. Samples for PESA were prepared on cleaned glass substrates. The thermal stability of **S10** and **S11** was investigated by TGA and DSC.

### 3.2. Cyclic-Voltammetry

CV was carried out in freshly distilled dichloromethane (over calcium hydride), with a supporting electrolyte of 0.1 M tetrabutylammoniumhexafluorophosphate (Bu_4_NPF_6_, Electrochemical grade, Aldrich), which was twice recrystallized from ethanol before use. A glassy carbon electrode was used as a working electrode (ALS, Tokyo, Japan), which was polished with 0.05 µM alumina on a felt pad, washed with distilled water followed by ethanol and dried under a N_2_ stream before use. A platinum wire was used as a counter electrode and a silver wire was used as a pseudo reference electrode. Ferrocene was used as an internal reference, by doping all solutions with an approximately equimolar amount of ferrocene. Reported voltammograms were recorded at a scan rate of 50 mV·s^−1^. Redox potentials (E_1/2_ values) were taken as a half-way point between forward and reverse peaks for each reversible redox process.

### 3.3. Device Preparation for Thin Film Transistors

Top contact/bottom gate OFET devices fabricated using *n*^+^-Si/SiO_2_ substrates where *n*^+^-Si and SiO_2_ work as gate electrode and gate dielectric, respectively. The thickness of thermally grown silicon oxide layer is around ~200 nm with a capacitance of about 17 nF/cm^2^. The SiO_2_/Si substrate was cleaned with acetone followed by isopropyl alcohol. It was then immersed in a piranha solution (V(H_2_SO_4_):V(H_2_O_2_) = 2:1) for 20 min, followed by rinsing with deionized water, and then immersed in a 0.1 M solution of hexamethyldisilazane (HMDS) in anhydrous toluene at 60 °C for 30 min. It was then rinsed with toluene followed by drying under nitrogen stream. Device fabrication was completed by deposition of **S10** and **S11** by spin coating CHCl_3_ solution (0.5 wt %) at 3000 rpm for 1 min. Subsequently, on top of the **S10** and **S11** active layer, a 100 nm thick gold (Au) thin film was deposited for source (S) and drain (D) electrodes through a shadow mask. For a typical OFET device reported here, the source-drain channel length (L) and channel width (W) was 100 μm and 1 mm, respectively. The device characteristics of the OFETs were measured at room temperature under nitrogen with a Keithley 4200 source meter. The field effect mobility (μ) was calculated from the saturation regime of transfer characteristics. Estimation of the carrier mobility was done using the standard transistor Equation (1) in saturation mode:

I_D_ = W/(2L)C_i_µ(V_G_ − V_T_)^2^ (1)

where μ is the field effect mobility, L is channel length, W is channel width, C_i_ is the gate insulator capacitance. 

### 3.4. Synthesis and Characterisation of Target Molecules

Both the materials, **S10** and **S11**, were synthesized by reacting the bis-bromoisoindigo precursor, (*E*)-6,6′-dibromo-1,1′-bis(2-ethylhexyl)-(3,3′-biindolinylidene)-2,2′-dione, at reflux, with 4-(diphenylamino)phenyl)boronic acid and carbazole in dimethoxyethane and toluene solvents, respectively. The reaction strategy is depicted in Scheme 1. Both the materials were purified through conventional column chromatography on silica and their chemical structures were confirmed via spectroscopic analyses.

*(E)-6,6′-bis(4-(Diphenylamino)phenyl)-1-(2-ethylheptyl)-1′-(2-ethylhexyl)-(3,3′-biindolinylidene)-2,2′-dione* (**S10**). (*E*)-6,6′-dibromo-1,1′-bis(2-ethylhexyl)-(3,3′-biindolinylidene)-2,2′-dione (0.25 g, 0.38 mmol) and (4-(diphenylamino)phenyl)boronic acid (0.27 g, 0.95 mmol) were mixed in dimethoxyethane (25 mL) in a 100 mL round-bottomed flask at room temperature and the reaction mixture was stirred for 15 min followed by the addition of cesium carbonate (Cs_2_CO_3_) (0.37 g, 1.14 mmol). The resulting suspension was degassed for 10 min by purging with argon, and tetrakis(triphenylphosphine)palladium(0) (Pd(PPh_3_)_4_) catalyst (0.10 g) was added to the reaction mixture. The reaction mixture was refluxed in an oil bath for 12 h in the absence of light and the progress of reaction was followed by thin layer chromatography, which indicated the consumption of starting dibromo derivative. The reaction mixture was cooled to room temperature, diluted with water (50 mL), and the product was extracted in chloroform. The organic layer was washed with water followed by brine, dried over anhydrous MgSO_4_, and recovered to get crude solid which was purified by column chromatography on silica (hexane: ethyl acetate 9:1 as eluent) to afford **S10** (0.26 g, 70%) as a bluish-black solid. IR (thin solid film, cm^−1^): 3034, 2957, 2928, 2858, 1693, 1608, 1591, 1518, 1493, 1455, 1357, 1330, 1281, 1179, 1109, 818, 754; ^1^H-NMR (400 MHz, CD_2_Cl_2_, TMS): δ (ppm) 9.22–9.19 (2H, m), 7.58–7.56 (4H, m), 7.32–7.25 (10H, m), 7.15–7.05 (16H, m), 7.04–7.00 (2H, m), 3.78–3.67 (4H, m), 1.97–1.86 (2H, m), 1.46–1.26 (16H, m), 0.97–0.93 (6H, m), 0.90–0.86 (6H, m); ^13^C-NMR (400 MHz, CD_2_Cl_2_, TMS): δ (ppm) 168.63, 148.13, 147.39, 145.87, 144.06, 133.71, 131.93, 129.95, 129.31, 127.56, 124.76, 123.55, 123.07, 120.53, 119.60, 105.78, 44.00, 37.69, 30.71, 28.72, 24.09, 23.05, 13.79, 10.47; HRMS (APCI): [M]^+^, found 972.5338. C_68_H_68_N_4_O_2_ requires 972.5337; Elemental Analysis for C_68_H_68_N_4_O_2_: Calculated C, 83.91; H, 7.04; N, 5.76; O, 3.29; Found C, 83.84; H, 7.01; N, 5.71; O, 3.27.

*(E)-6,6′-di(9H-Carbazol-9-yl)-1-(2-ethylheptyl)-1′-(2-ethylhexyl)-(3,3′-biindolinylidene)-2,2′-dione* (**S11**). (*E*)-6,6′-dibromo-1,1′-bis(2-ethylhexyl)-(3,3′-biindolinylidene)-2,2′-dione (0.25 g, 0.38 mmol) was added to the mixture of carbazole (0.15 g, 0.87 mmol), sodium *t*-butoxide (0.11 g, 1.14 mmol), and (1,1′-bis(diphenylphosphino)ferrocene)dichloropalladium(II), complex with dichloromethane (Pd(dppf)Cl_2_∙CH_2_Cl_2_) (0.081 g, 0.1 mmol) in toluene (25 mL) followed by the addition of 1,1′-bis(diphenylphosphino)ferrocene (0.22 g, 0.4 mmol) at room temperature, and the resulting suspension was refluxed overnight. The reaction mixture was cooled to room temperature, filtered through Celite bed and the solvent was evaporated off to get crude solid, which was subjected to column chromatography on silica (10% ethyl acetate/hexane) to afford title compound **S11** (0.161 g, 52%) as a black solid. IR (thin solid film, cm^−1^): 3057, 2958, 2928, 2872, 1694, 1610, 1501, 1447, 1384, 1334, 1224, 1112, 1099, 736; ^1^H NMR (400 MHz, CD_2_Cl_2_, TMS): δ (ppm) 9.50–9.48 (2H, m), 8.18–8.16 (4H, m), 7.63–7.61 (4H, m), 7.49–7.45 (4H, m), 7.35–7.31 (6H, m), 7.10–7.09 (2H, m), 3.80–3.66 (4H, m), 1.97–1.87 (2H, m), 1.48–1.22 (16H, m), 0.96–0.92 (6H, m), 0.86–0.82 (6H, m); ^13^C-NMR (400 MHz, CD_2_Cl_2_, TMS): δ (ppm) 168.50, 146.88, 141.07, 140.21, 132.19, 131.23, 126.17, 123.76, 120.48, 120.31, 120.21, 119.28, 110.20, 106.12, 44.38, 37.81, 30.74, 28.74, 24.04, 23.00, 13.74, 10.41; HRMS (APCI): [M]^+^, found 816.4397. C_56_H_56_N_4_O_2_ requires 816.4398; Elemental Analysis for C_56_H_56_N_4_O_2_: Calculated C, 82.32; H, 6.91; N, 6.86; O, 3.92; Found C, 82.27; H, 6.86; N, 6.79; O, 3.89.

## 4. Conclusions

In conclusion, we have demonstrated the use of isoindigo accepting functionality to generate new D–A–D modular small organic molecules, **S10** and **S11**, which contain a common isoindigo core unit and varied donor functionalities. The new materials, **S10** and **S11**, were synthesized, found to be highly soluble in a variety of common organic solvents, and were thermally stable. Use of a TPA unit as an energy antenna helped to achieve a substantial red-shift of the absorption maximum in the visible region, improved the solubility of target material and helped to reduce optical band-gap. Upon testing these materials as active layers in OFET devices, hole mobilities of the order of 2.2 × 10^−4^ cm^2^/Vs and 7.8 × 10^−3^ cm^2^/Vs were achieved for **S10** and **S11**, respectively. There are several reports on isoindigo-based polymers but not much work has been done so far on the design and development of small molecules. The reported charge-carrier mobility values for **S10** and **S11** are promising and through further visionary designing, high mobility values can be achieved. Our results on the effect of donor types on electronic properties of D–A–D modular organic materials can indeed inform the design of futuristic materials for organic electronic applications. Future studies will focus on the use of unsymmetrical donor units and incorporation of π-spacers between donor and acceptor functionalities.

## Supplementary Materials

Supplementary materials can be accessed at: http://www.mdpi.com/1420-3049/20/09/17362/s1.

## References

[1] Wu H., Ying L., Yang W., Cao Y. (2009). Progress and perspective of polymer white light-emitting devices and materials. Chem. Soc. Rev..

[2] Pron A., Gawrys P., Zagorska M., Djurado D., Demadrille R. (2010). Electroactive materials for organic electronics: Preparation strategies, structural aspects and characterisation techniques. Chem. Soc. Rev..

[3] Hadfield R.H. (2009). Single-photon detectors for optical quantum information applications. Nat. Photon..

[4] Cheng Y.J., Yang S.H., Hsu C.S. (2009). Synthesis of conjugated polymers for organic solar cell applications. Chem. Rev..

[5] Gupta A., Watkins S.E., Scully A.D., Singh T.B., Wilson G.J., Rozanski L.J., Evans R.A. (2011). Band-gap tuning of pendant polymers for organic light-emitting devices and photovoltaic applications. Synth. Met..

[6] Helgesen M., Søndergaard R., Krebs F.C. (2012). Advanced materials and processes for polymer solar cell devices. J. Mater. Chem..

[7] Li Y., Guo Q., Li Z., Pei J., Tian W. (2010). Solution processable D–A small molecules for bulk-heterojunction solar cells. Energ. Environ. Sci..

[8] Mishra A., Bäuerle P. (2012). Small molecule organic semiconductors on the move: Promises for future solar energy technology. Angew. Chem. Inter. Ed..

[9] Rybakiewicz R., Djurado D., Cybulski H., Dobrzynska E., Kulszewicz-Bajer I., Boudinet D., Verilhac J.-M., Zagorska M., Pron A. (2011). Arylene bisimides with triarylamine *N*-substituents as new solution processable organic semiconductors: Synthesis, spectroscopic, electrochemical and electronic properties. Synth. Met..

[10] Marks T.J., Ratner M.A. (1995). Design, synthesis, and properties of molecule-based assemblies with large second-order optical nonlinearities. Angew. Chem. Int. Ed..

[11] Marder S.R., Kippelen B., Jen A.K.Y., Peyghambarian N. (1997). Design and synthesis of chromophores and polymers for electro-optic and photorefractive applications. Nature.

[12] Facchetti A. (2011). π-Conjugated polymers for organic electronics and photovoltaic cell applications. Chem. Mater..

[13] Günes S., Neugebauer H., Sariciftci N.S. (2007). Conjugated polymer-based organic solar cells. Chem. Rev..

[14] Lin Y., Li Y., Zhan X. (2012). Small molecule semiconductors for high-efficiency organic photovoltaics. Chem. Soc. Rev..

[15] Gupta A., Ali A., Bilic A., Gao M., Hegedus K., Singh T.B., Watkins S.E., Wilson G.J., Bach U., Evans R.A. (2012). Absorption enhancement of oligothiophene dyes through the use of a cyanopyridone acceptor group in solution-processed organic solar cells. Chem. Commun..

[16] Gupta A., Ali A., Singh B., Bilic A., Bach U., Evans R.A. (2012). Molecular engineering for panchromatic absorbing oligothiophene donor-π-acceptor organic semiconductors. Tetrahedron.

[17] Gupta A., Armel V., Xiang W., Fanchini G., Watkins S.E., MacFarlane D.R., Bach U., Evans R.A. (2013). The effect of direct amine substituted push–pull oligothiophene chromophores on dye-sensitized and bulk heterojunction solar cells performance. Tetrahedron.

[18] Kumar R.J., Churches Q.I., Subbiah J., Gupta A., Ali A., Evans R.A., Holmes A.B. (2013). Enhanced photovoltaic efficiency via light-triggered self-assembly. Chem. Commun..

[19] Patil H., Gupta A., Bilic A., Jackson S.L., Latham K., Bhosale S.V. (2014). Donor–acceptor–donor modular small organic molecules based on the naphthalenediimide acceptor unit for solution-processable photovoltaic devices. J. Electron. Mater..

[20] Tamayo A.B., Dang X.-D., Walker B., Seo J., Kent T., Nguyen T.-Q. (2009). A low band gap, solution processable oligothiophene with a dialkylated diketopyrrolopyrrole chromophore for use in bulk heterojunction solar cells. Appl. Phys. Lett..

[21] Walker B., Tamayo A.B., Dang X.-D., Zalar P., Seo J.H., Garcia A., Tantiwiwat M., Nguyen T.-Q. (2009). Nanoscale phase separation and high photovoltaic efficiency in solution-processed, small-molecule bulk heterojunction solar cells. Adv. Funct. Mater..

[22] Li Z., Dong Q., Li Y., Xu B., Deng M., Pei J., Zhang J., Chen F., Wen S., Gao Y. (2011). Design and synthesis of solution processable small molecules towards high photovoltaic performance. J. Mater. Chem..

[23] Shi Q., Cheng P., Li Y., Zhan X. (2012). A solution processable D-A-D molecule based on thiazolothiazole for high performance organic solar cells. Adv. Energy. Mater..

[24] Stalder R., Mei J., Reynolds J.R. (2010). Isoindigo-based donor-acceptor conjugated polymers. Macromolecules.

[25] Zhang G., Fu Y., Xie Z., Zhang Q. (2011). Synthesis and photovoltaic properties of new low bandgap isoindigo-based conjugated polymers. Macromolecules.

[26] Lei T., Cao Y., Fan Y., Liu C.-J., Yuan S.-C., Pei J. (2011). High-performance air-stable organic field-effect transistors: Isoindigo-based conjugated polymers. J. Am. Chem. Soc..

[27] Stalder R., Mei J., Graham K.R., Estrada L.A., Reynolds J.R. (2014). Isoindigo, a versatile electron-deficient unit for high performance organic electronics. Chem. Mater..

[28] Deng P., Zhang Q. (2014). Recent developments on isoindigo-based conjugated polymers. Polym. Chem..

[29] Lei T., Wang J.-Y., Pei J. (2014). Design, synthesis, and structure-property relationships of isoindigo-based conjugated polymers. Acc. Chem. Res..

[30] Tian H., Yang X., Cong J., Chen R., Liu J., Hao Y., Hagfeldt A., Sun L. (2009). Tuning of phenoxazine chromophores for efficient organic dye-sensitized solar cells. Chem. Commun..

[31] Frisch M.J., Trucks G.W., Schlegel H.B., Scuseria G.E., Robb M.A., Cheeseman J.R., Scalmani G., Barone V., Mennucci B., Petersson G.A. (2013). Gaussian 09.

[32] Dasari R.R., Dindar A., Lo C.K., Wang C.-Y., Quinton C., Singh S., Barlow S., Reynolds J.R., Fuentes-Hernandez C., Kippelen B. (2014). Tetracyano isoindigo small molecules and their use in *n*-channel organic field-effect transistors. Phys. Chem. Chem. Phys..

[33] McEwen C.N., McKay R.G., Larsen B.S. (2007). Analysis of solids, liquids, and biological tissues using solids probe introduction at atmospheric pressure on commercial LC/MS instruments. Anal. Chem..

