# Supplementary material for: Isoindigo-Based Small Molecules with Varied Donor Components for Solution-Processable Organic Field Effect Transistor Devices
Source: Molecules. 2015 Sep 18;20(9):17362–77. doi: 10.3390/molecules200917362 (PMC6332248; doi:10.3390/molecules200917362)
Supplement: Supplementary file 1 [file molecules-20-17362-s001.pdf]

Supplementary Materials

S10

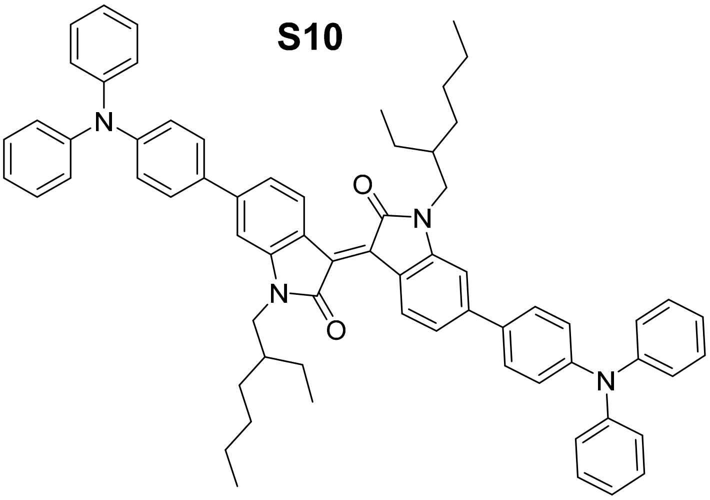

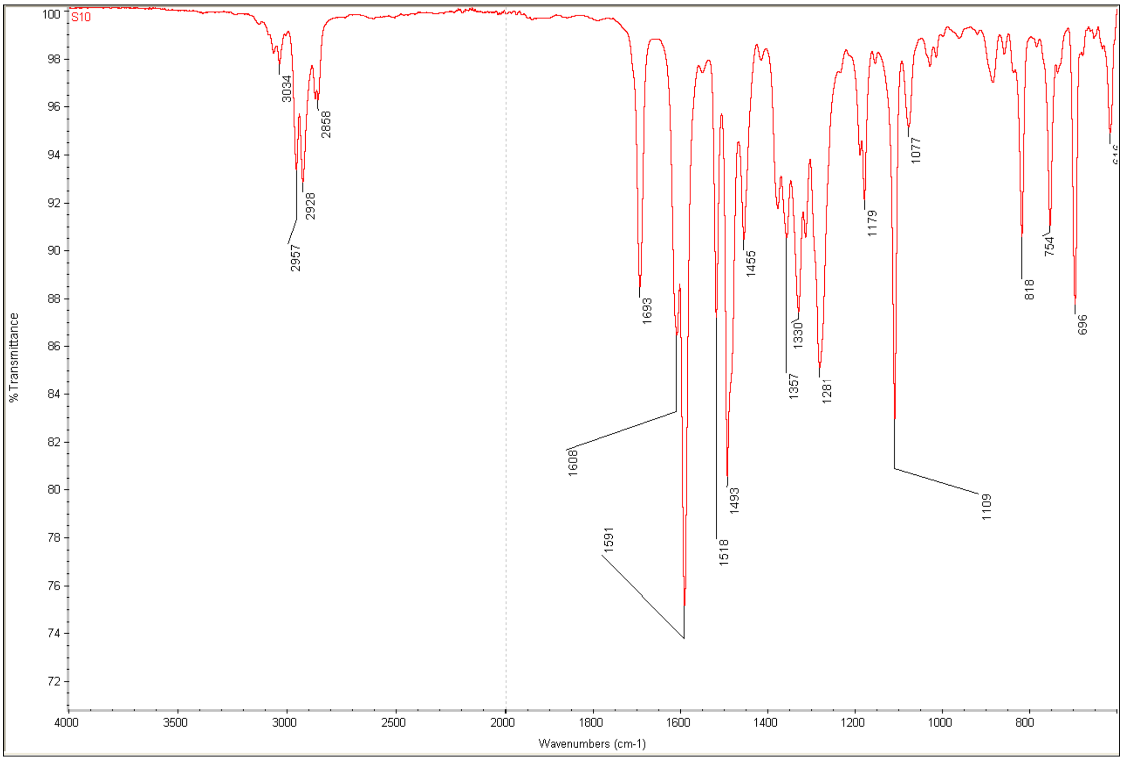

Figure S1. Infrared (IR) spectrum of **S10**.

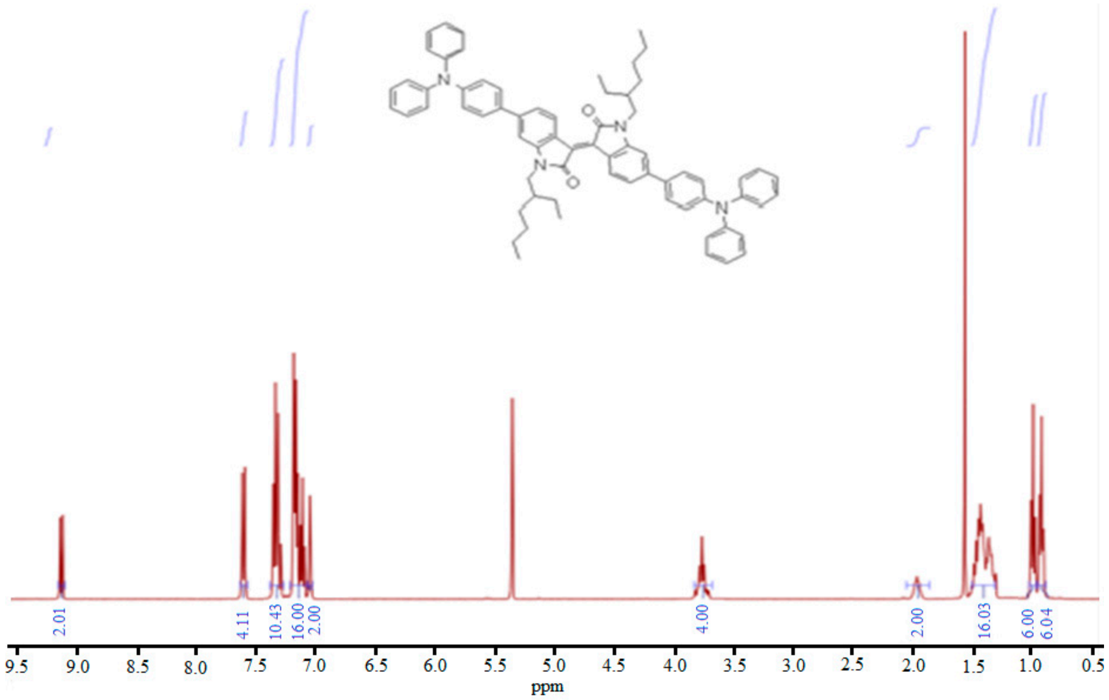

Figure S2. ^{1}H-NMR spectra of **S10**.

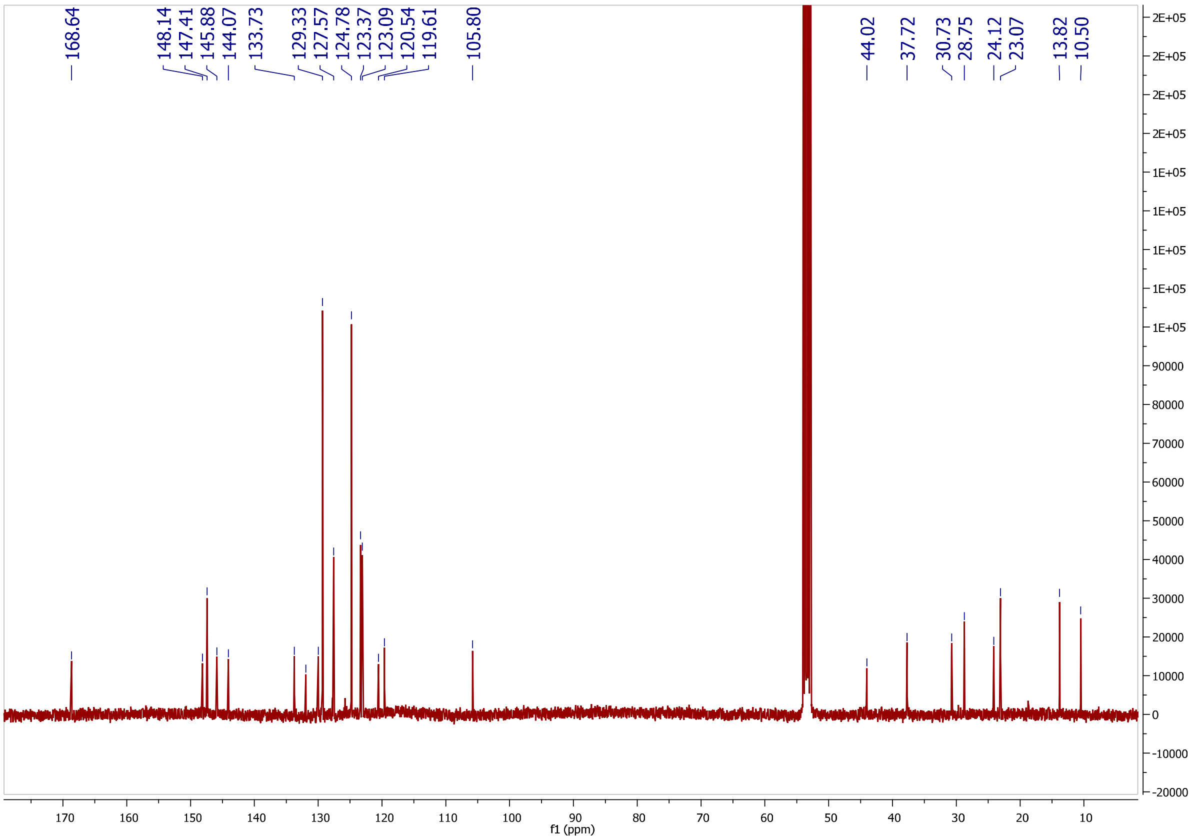

Figure S3. ^{13}C-NMR (below) spectra of **S10**.

CSIRO Material Science & Engineering
A.Gupta

QExactive ASAP Probe Accurate Mass Report
S10

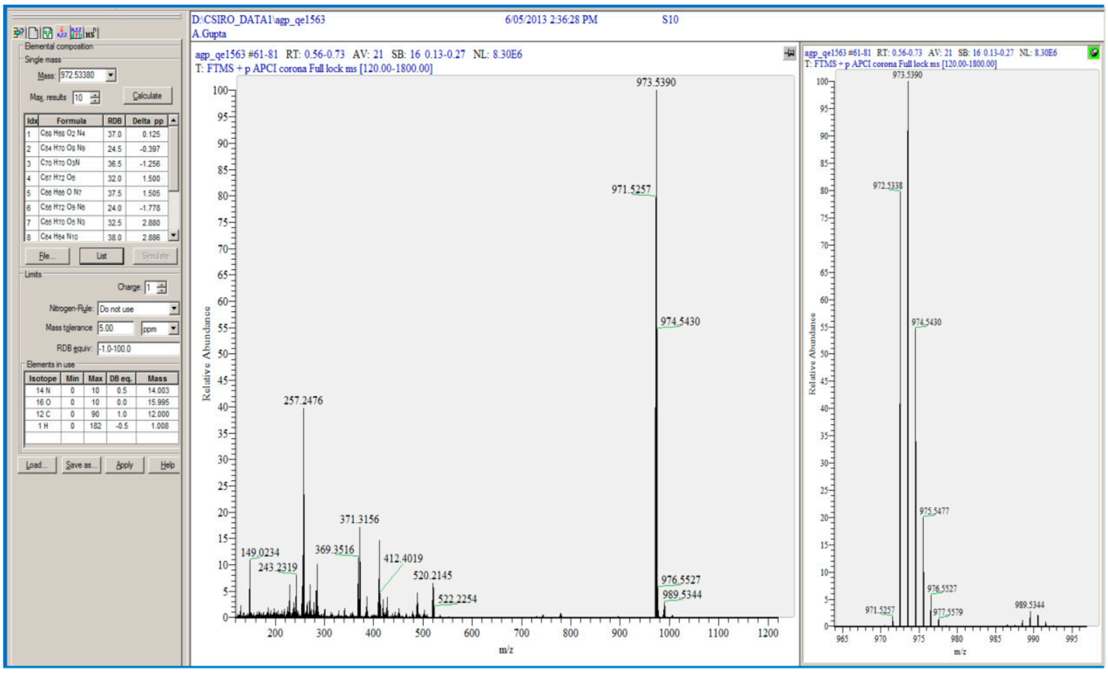

All experiments were carried out on a Thermo Scientific Q Exactive FTMS, employing ASAP probe.

1 October 2013 Page 1 of 2

CSIRO Material Science & Engineering
A.Gupta

QExactive ASAP Probe Accurate Mass Report
S10

m/z	Theo. Mass	Delta (ppm)	Composition
972.5338	972.5337	0.12	C68 H68 O2 N4
	972.5342	-0.40	C54 H70 O8 N9
	972.5350	-1.26	C70 H70 O3 N
	972.5323	1.50	C67 H72 O6
	972.5323	1.51	C66 H66 O N7
	972.5355	-1.78	C56 H72 O9 N6
	972.5310	2.88	C65 H70 O5 N3
	972.5310	2.89	C64 H64 N10
	972.5369	-3.15	C57 H68 O5 N10
	972.5369	-3.16	C58 H74 O10 N3

All experiments were carried out on a Thermo Scientific Q Exactive FTMS, employing ASAP probe.

1 October 2013 Page 2 of 2

Figure S4. HRMS spectrum of **S10**.

S11

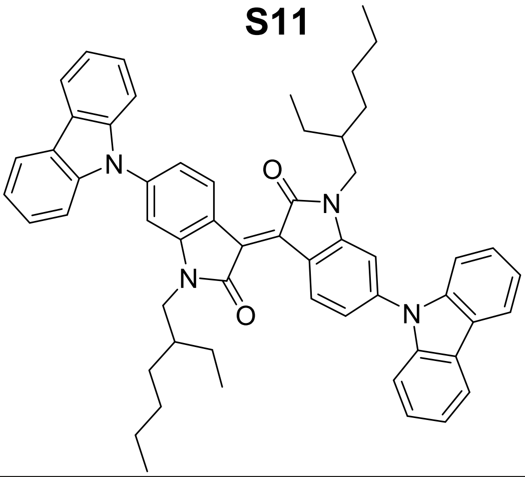

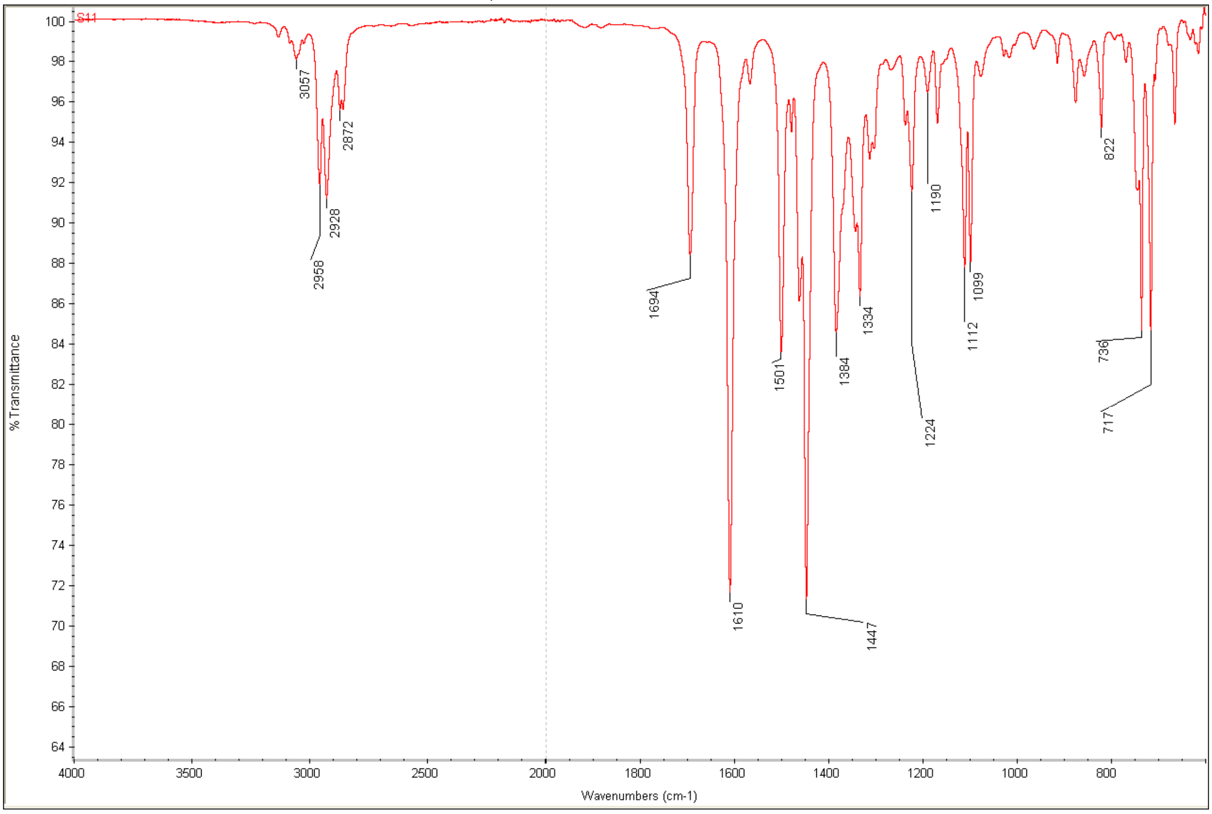

Figure S5. IR spectrum of **S11**.

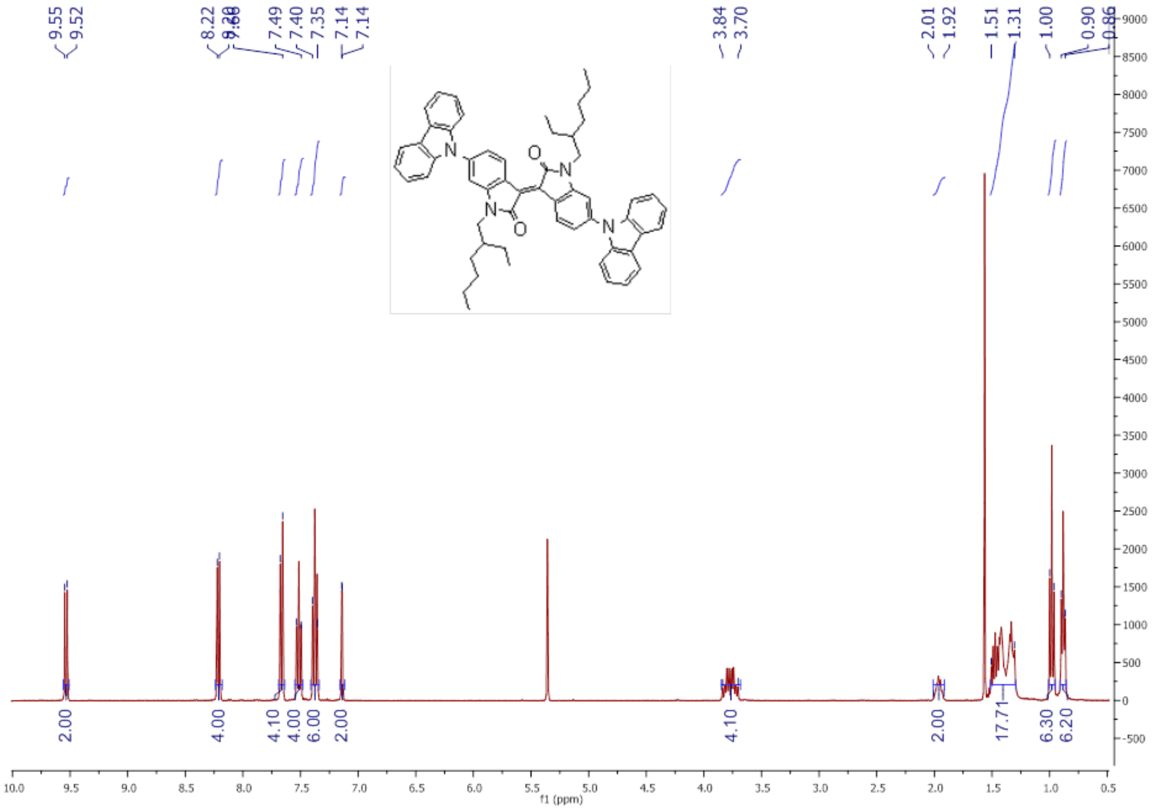

Figure S6. ^{1}H-NMR spectra of **S11**.

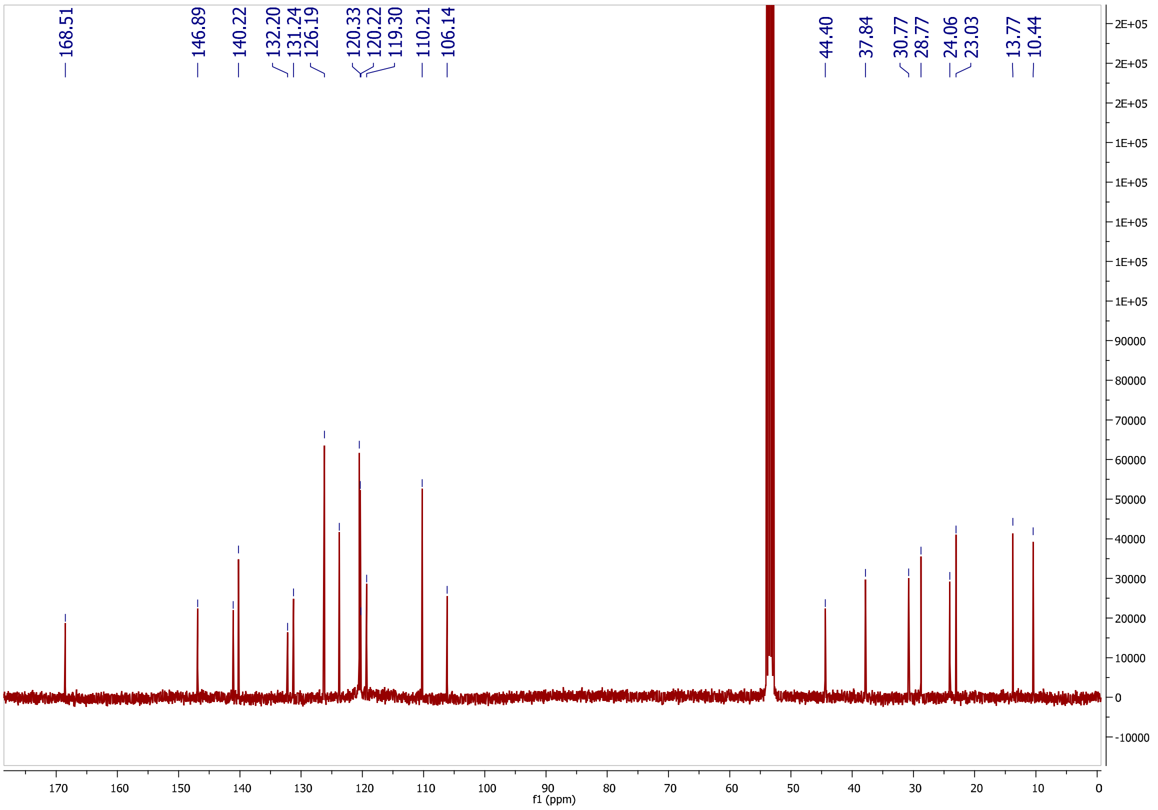

Figure S7. ^{13}C-NMR spectra of **S11**.

CSIRO Material Science & Engineering
A.Gupta

QExactive ASAP Probe Accurate Mass Report
S11

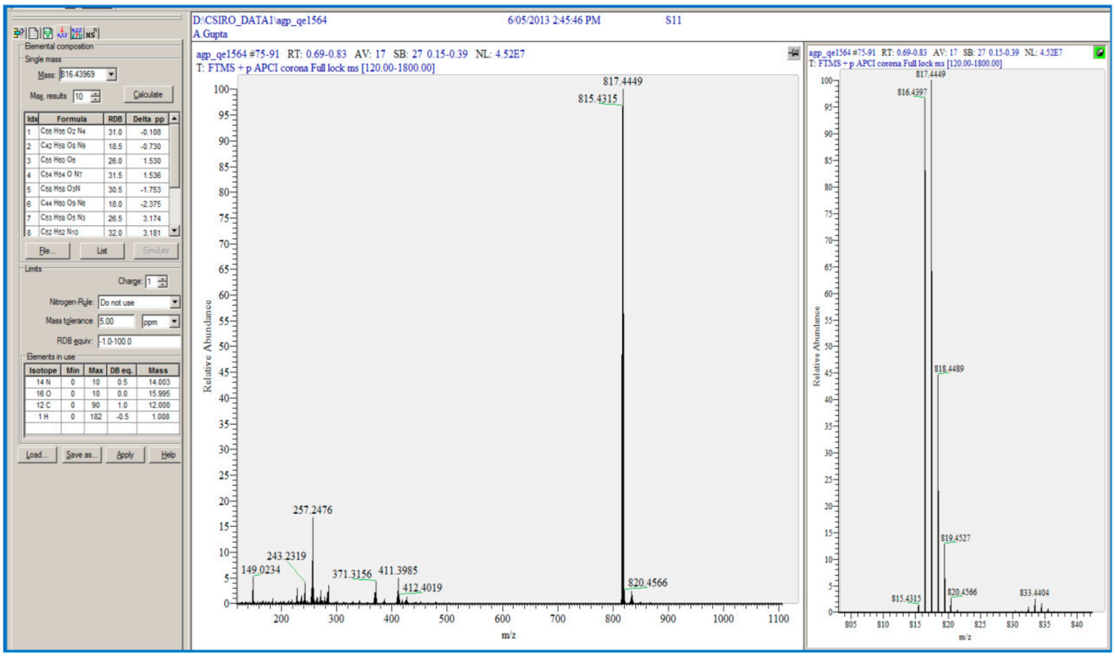

All experiments were carried out on a Thermo Scientific Q Exactive FTMS, employing ASAP probe.

CSIRO Material Science & Engineering
A.Gupta

QExactive ASAP Probe Accurate Mass Report
S11

m/z	Theo. Mass	Delta (ppm)	Composition
816.4397	816.4398	-0.11	C56 H56 O2 N4
	816.4403	-0.73	C42 H58 O8 N9
	816.4384	1.53	C55 H60 O6
	816.4384	1.54	C54 H54 O N7
	816.4411	-1.75	C58 H58 O3 N
	816.4416	-2.37	C44 H60 O9 N6
	816.4371	3.17	C53 H58 O5 N3
	816.4371	3.18	C52 H52 N10
	816.4430	-4.01	C45 H56 O5 N10
	816.4430	-4.02	C46 H62 O10 N3

All experiments were carried out on a Thermo Scientific Q Exactive FTMS, employing ASAP probe.

Figure S8. HRMS spectrum of **S11**.

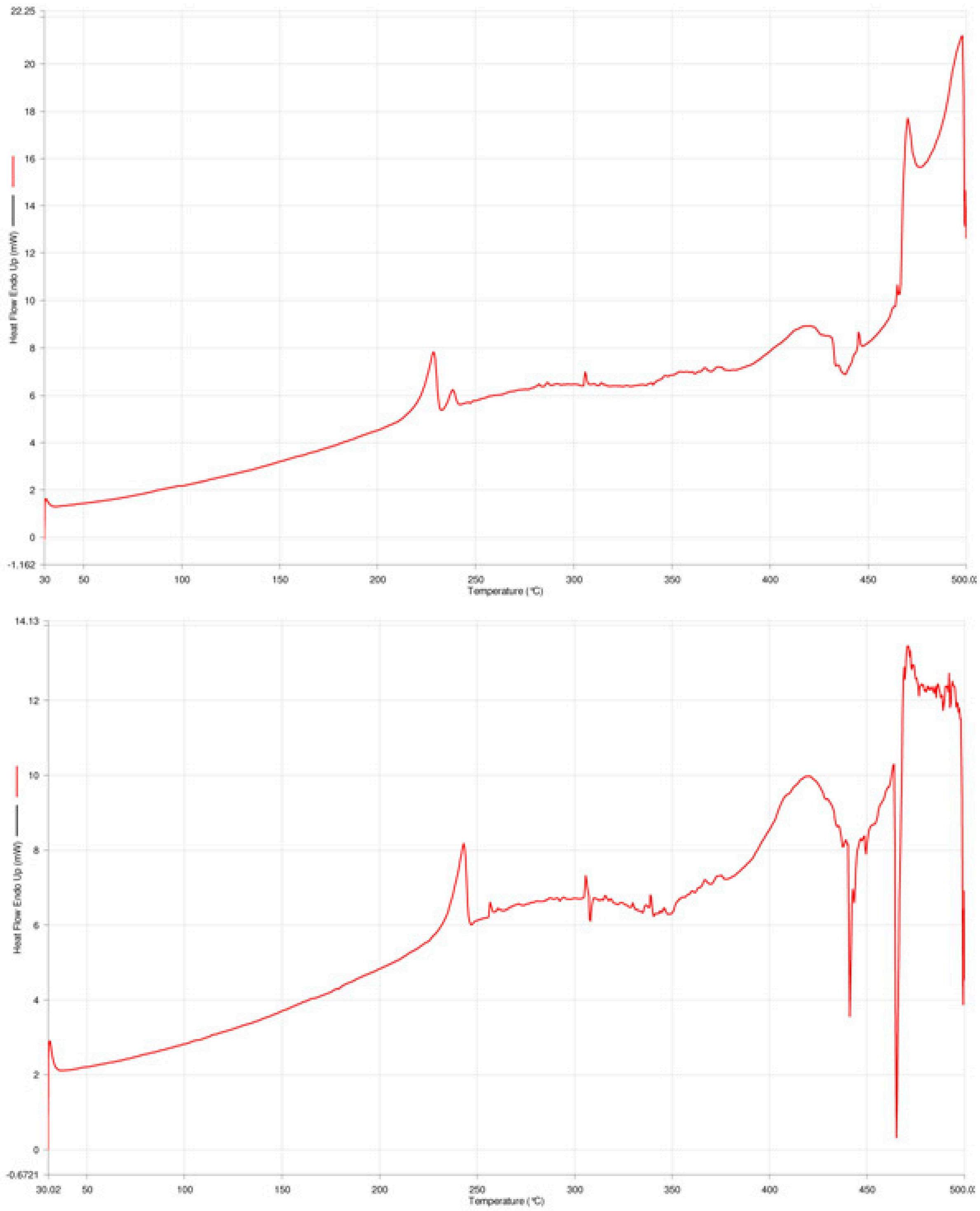

Figure S9. DSC curves of **S10** (above) and **S11** (below).

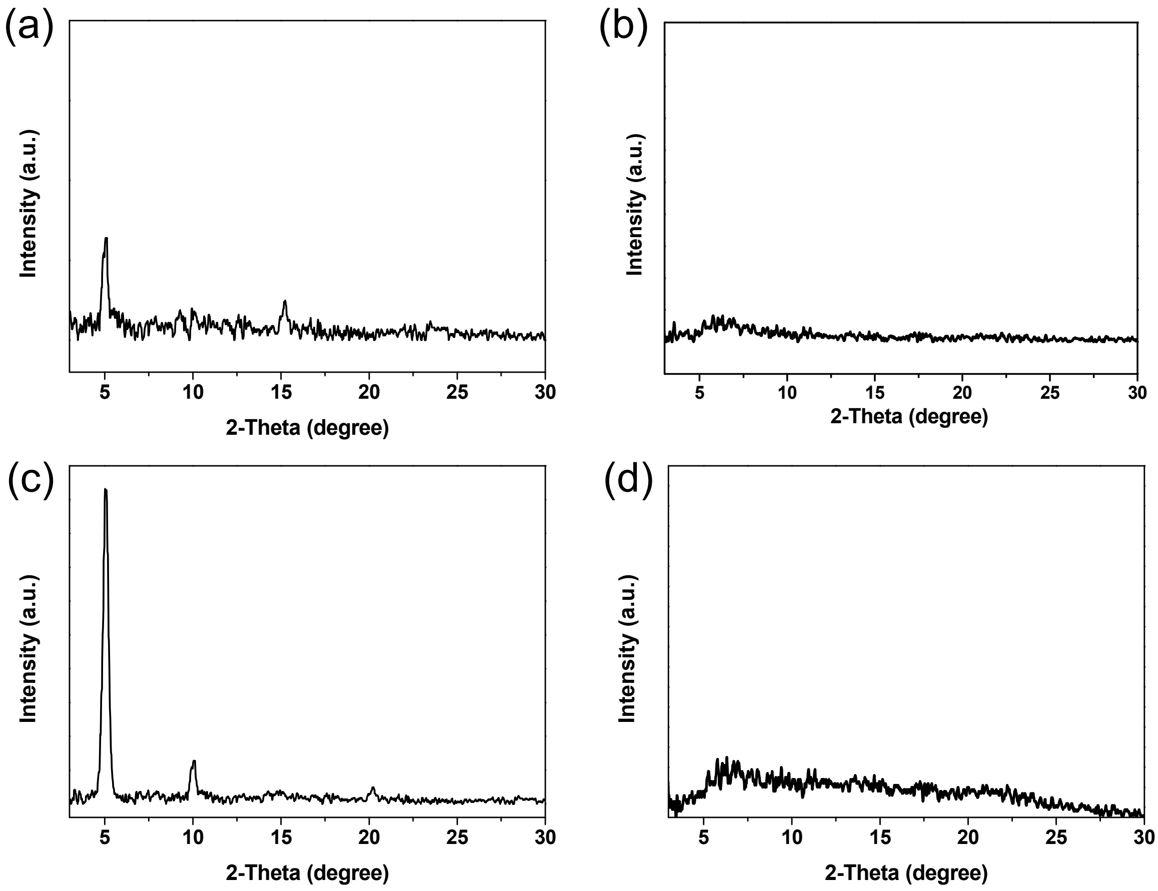

Figure S10. The XRD spectra of **S10-** (**a**,**c**) and **S11**-based (**b**,**d**) thin films under as-spun (**a**,**b**) and thermal annealing at 120 °C (**c**,**d**) conditions.
